# Socio-demographic factors associated with mental health disorders among rural women in Mymensingh, Bangladesh

**DOI:** 10.3389/fpsyt.2025.1446473

**Published:** 2025-02-18

**Authors:** Rizwana Khan, Fahmida Akter, Musarrat Rahman, Nuhu Amin, Mahbubur Rahman, Peter J. Winch

**Affiliations:** ^1^ Infectious Disease Division, International Center for Diarrheal Disease Research, Dhaka, Bangladesh; ^2^ Arnold School of Public Health, University of South Carolina, Columbia, SC, United States; ^3^ Department of International health, John Hopkins Bloomberg School of Public Health, Baltimore, MD, United States; ^4^ Health Systems and Population Studies Division, International Center for Diarrheal Disease Research, Dhaka, Bangladesh

**Keywords:** socio-demographic status, rural women, mental health, global burden of disease, Bangladesh

## Abstract

**Introduction:**

In Bangladesh, the reported prevalence of mental disorders among adults varies from 6.5% to 31.0%. This study aims to identify the socio-demographic factors associated with mental health disorders among rural women in Bangladesh.

**Method:**

We enrolled 401 adult women from four sub-districts of Mymensingh district, Bangladesh. To determine the factors involved, we employed a modified version of the mental state examination (MSE) scale, in addition to questionnaires focusing on socio-demographic information, health, and well-being.

**Result:**

The prevalence of mental health problems, measured by the MSE scale, was 26%. Even after controlling for potential confounders such as respondent's age, education level, spouse's schooling, household income, health status, domestic violence, family disharmony, social security, and support, household income remained significantly associated with behavioral problems. Individuals with higher incomes experience a lower prevalence of mental health disorders (Prevalence Ratio [PR] = 0.48; 95% CI 0.27-0.86). Poor physical health significantly correlated with behavior and mood (PR = 0.77; 95% CI 0.61-0.98). Older respondents encountered more challenges related to memory (PR = 1.42; 95% CI 1.13-1.79).

**Discussion:**

These findings highlight the crucial role of addressing socio-demographic factors, like income and physical health, when promoting mental health among rural women in Bangladesh. By acknowledging and targeting these factors, interventions can be more tailored and effective, ensuring improved well-being and resilience within this population.

## 1 Introduction

### 1.1 Mental health in low- and middle-income countries

Mental health functioning is essential for human well-being, encompassing emotional regulation, cognitive abilities, and the capacity to manage daily responsibilities. Mental disorders significantly contribute to the global burden of non-communicable diseases (NCDs) (1, 2). They profoundly impact social, economic, and physical health outcomes, with impairments leading to reduced productivity, strained interpersonal relationships, and diminished quality of life. Despite its critical importance, mental health remains one of the most neglected areas in global public health, contributing to 13% of the global burden of disease (3) This burden is disproportionately high in low- and middle-income countries (LMICs), which account for 75% of global mental health cases (4).

The societal and economic impact of mental health disorders is substantial. In 2017, mental disorders ranked as the second leading contributor to the global disease burden in terms of years lived with disability (YLDs) and the sixth leading cause of disability-adjusted life years (DALYs), particularly in low and middle-income countries (LMICs) (5).

The worldwide prevalence of common mental disorders is 17.6%, with LMICs experiencing an even higher prevalence of 22.7% (6, 7). Alarmingly, one in four individuals globally is projected to experience a mental illness during their lifetimes (8–10). Despite these figures, mental health services remain underfunded and underdeveloped, resulting in significant gaps in care (11, 12).

The treatment gap, which refers to the disparity between individuals needing mental health care and those receiving it, is particularly concerning in LMICs, where up to 90% of cases go untreated (13). This gap is driven by inadequate healthcare infrastructure, a shortage of trained mental health professionals, cultural stigma, and low mental health literacy (14) Vulnerable groups, particularly women in rural areas, face heightened barriers due to socio-economic disadvantages, restricted autonomy, and limited access to resources (15, 16). Inadequate access to mental health care, individuals struggle to maintain employment, relationships, and overall well-being (17). Undiagnosed mental health conditions can drastically shorten life expectancy (18).

### 1.2 Mental health in rural Bangladesh

Mental health issues are often overshadowed by communicable diseases, despite their high occurrence (19–22). Bangladesh faces a significant treatment gap for mental disorders, with 91% of cases going untreated (23). This challenge is further compounded by the absence of a dedicated and comprehensive mental health policy. Previous policies and action plans have addressed mental health disorders as part of broader non-communicable disease initiatives. However, this approach has proven inadequate to provide effective care for the prevention, diagnosis, treatment, and rehabilitation of mental health disorders, especially in a densely populated country like Bangladesh (24). A national mental health survey conducted between 2003 and 2005 revealed that 16.1% of the elderly population in Bangladesh suffered from mental disorders, a figure that rose to 18.7% by 2019 (8, 25), highlighting the need for better treatment options and support in public healthcare.

Bangladesh, where over 60% of the population resides in rural areas, exemplifies these challenges. Rural women in Bangladesh are particularly vulnerable to poor mental health due to factors such as low educational attainment, limited financial resources, food insecurity, and exposure to domestic violence (26). Approximately 20% of rural Bangladeshi women are reported to suffer from major depressive disorder, with many experiencing impairments in mood, attention, and memory (27). Food insecurity, which affects nearly 40% of rural households, further exacerbates these mental health challenges (28).

Mental health services in Bangladesh remain critically under-resourced. With only 0·073 psychiatrists available per 100,000 people, mental health receives just 0.44% of the national health budget (8). Most mental health services are concentrated in urban centers, leaving rural populations without adequate access to care (29). Moreover, stigma surrounding mental illness often prevents individuals, especially women, from seeking help, further widening the treatment gap (30).

The economic and societal repercussions of untreated mental health issues are profound. Globally, the economic cost of mental disorders is projected to reach $16 trillion by 2030 (12). Impairments in mental health functioning hinder women’s ability to manage household responsibilities, care for their families, and engage in economic activities (16). Additionally, the interdependence between mental and physical health perpetuates a cycle of poor health outcomes, particularly for rural women (27). Addressing this urgent issue requires increased investment in mental health services and resources, aligning with the Sustainable Development Goals (SDGs) which emphasize the prevention of mental disorders and promotion of mental well-being (12).

Given the significant burden of mental health issues in LMICs, particularly in rural Bangladesh, there is a pressing need to investigate the socio-demographic determinants of mental health functioning in this population. Moreover, epidemiological and health system data on mental disorders in Bangladesh are limited and not easily accessible, although a few published studies offer some estimates of various mental health conditions. This study aims to bridge this research gap by exploring the social and demographic factors contributing to mental health issues among rural women in Bangladesh. Using a modified Mental State Examination (MSE) scale, the study evaluates key neurobehavioral domains to identify patterns of impairments and their socio-demographic correlates. The findings will contribute to evidence-based, culturally tailored interventions and inform policies to address the unique mental health needs of rural Bangladeshi women.

## 2 Methods

### 2.1 Study design

We conducted a cross-sectional study in the rural areas of Shomvugong, Ishwargong, Fulpur, and Tarakanda sub-districts in Mymensingh district, Bangladesh. After obtaining approval from the Institutional Review Board, the data collection took place over three months, from August 15 to November 15, 2015.

### 2.2 Sample size and sampling

The prevalence of mental disorders ranges from 6.5% to 31% (6), and the total population of four subdistricts was approximately 1.4 million (31), the following formula for sample size calculation in a population survey:

- n = Z^2^ × p × (1-p)/E^2^
- n is the required sample size
- Z is the Z-score corresponding to the desired confidence level (e.g., 1.96 for 95% confidence)
- p is the estimated prevalence (31% or 0.31)

E is the margin of error (0.05 or 5%)95% confidence level and a 5% margin of error (0.05), the sample size calculation is:


$$\begin{array}{c} n={\left(1.96\right)}^{2}\times 0.31\\ \times \left(1-0.31\right)/0.05^{2}\text{n}=3.8416\times 0.31\times 0.69/0.0025 \end{array}$$

n=0.8235/0.0025=329.4

n=330

Given the estimated approximate population size the calculated sample size based on the prevalence of 31% was 330 participants. However, to account for potential non-responses, dropouts, and other unforeseen factors, we opted to increase the sample size to 401. This adjustment ensures that the study retains statistical power and remains representative, even of incomplete data or participant attrition. Selecting a slightly larger sample provides more robust results while mitigating sampling bias and minimizing the potential loss of data.

The study site was the Mymensingh district of central Bangladesh (in the 2011 census) which consists of 12 subdistricts (31). For this study, we selected four subdistricts of Mymensingh district based on the selection criteria such as i) Good road communication ii) not a hard-to-reach area iii) rural settings, and iv) no similar ongoing study. We followed a multistage sampling strategy to select households for inclusion in the survey. In each subdistrict, a complete list of all villages was obtained from administrative records and the average number of villages ranged from 120 to 225 in each subdistrict. We followed A random selection of ten villages from each list using the random number generation method in Excel. At the end of the process, we selected 40 villages from the four subdistricts. We applied a systematic sampling strategy to enroll households from the selected villages. In this process, we first identified the center of each village as reported by the local residents. From the chosen center of the village, we walked in a straight line after spinning a pen to determine the direction to walk and then we went to every third eligible household until 10 households were identified. The eligibility criteria for households were i) at least one resident who was woman aged 18-65 years and ii) No visual, hearing, or other significant cognitive disabilities. In the eligible households, we recruited the participants who provided informed consent to participate in the study. In case of refusal, we replaced the households with the next eligible ones. We recruited and consented participants until we had enrolled total of 401 women from the four sub-districts. After enrolling the required number of women in each sub-district, we collected data through structured interviews and questionnaires.

### 2.3 Pilot study

A semi-structured questionnaire was prepared aligned with the study goals and incorporating relevant scales. We piloted the questionnaire in Mymensingh Sadar a separate sub-district within the same district and involved 20 women to ensure that the scale or tool accurately and reliably measures what it is intended to measure. However, the pilot test was done to reword questions, adjust response options, and modify the structure. This preliminary testing provided valuable insights, leading to refinements that improved the tool’s clarity and effectiveness before full implementation.

Based on the piloting results, necessary changes were made and finalized before initiating data collection.

### 2.4 Conduct of the study, quality control, and monitoring

Before fieldwork, the supervisor a psychiatrist and public health professional, conducted a comprehensive 7-day training session at the National Institute of Preventive and Social Medicine (NIPSOM). The training sessions include detailed data collection procedures and orientation on the Mental Status Examination (MSE) scale. The training sessions were followed by lectures, role-playing and dummy practice in the classroom, field practice, iterative reviews, and feedback to equip the investigator for field implementation. Topics included mental health concepts, study objectives, informed consent and ethical consideration, data collection techniques and tools, sampling strategies, interview methods, safety protocols, etc. role-playing, dummy exercises, and field practices made confident and improved the skillsets of trainee to ensure quality data collection. The training ensured quality assurance, ethical adherence, and alignment with the study protocol, culminating in the finalization of the questionnaire for field testing and data collection. The supervisor’s engagement ensured the capacity and quality of training, the building of confidence in the field implementation of tools, and further analysis. Through this process, the supervisor was able to improve the capacity and transfer knowledge and skillsets to the trainee to enable field implementation of tools and further development.

The investigator, a physician by training, fully understood the importance of these guidelines and diligently followed them throughout the entire data collection process. The investigator was able to create an environment where the participants felt at ease and comfortable enough to openly share their experiences. During data collection, the investigator conducted approximately five interviews per day. After each working day, the collected data were reviewed and verified. Any inaccuracies or inconsistencies were corrected.

### 2.5 MSE scale

The Mental State Examination (32) is a broader assessment tool used in psychiatric evaluations to assess a patient’s mental status (33, 34), including an essential component of the clinical assessment process in psychiatric practice. It typically involves a clinician’s subjective assessment based on observation and interaction with the patient. It provides a structured approach to observing and describing a patient’s current mental state across different domains, including appearance, attitude, behavior, mood and affects, speech, thought process, thought content, perception, cognition, insight, and judgment. While slight variations in the organization and terminology used for the MSE’s subdivisions and domains may exist, its overall purpose remains consistent.

We implemented a revised edition of the Mental State Examination (35) scale and supplementary surveys covering socio-demographic and social influencing factors. The subscales encompassed specific neurobehavioral domains (Attitude, Sleep, Attention, and Memory) (36) in addition to the primary domains (Appearance, Behavior, Speech, Mood, Insight, and Judgment) of the MSE scale. To address the validity of the tailored tool, we retained the core components of the MSE that align with the study’s objectives, focusing on observable and measurable aspects such as mood, appearance, behavior and so forth. The MSE is a flexible tool designed to be adapted based on the specific objectives, context of the assessment, and the expertise of the examiner (37). The omitted components, such as Perception, Thought, and Cognition, were not integral to our primary objectives. Instead, the retained components were interpreted independently (38, 39) and systematically, ensuring that each domain provided meaningful insights. Certain components (Perception, Thought, Cognition) were omitted as they necessitate evaluation by a licensed psychiatrist (40). This tailored version of the MSE scale enabled us to effectively evaluate the mental state of our subjects while upholding precision. By concentrating on the essential elements, we obtained valuable insights without overwhelming our limited resources (**Table 1**).

**Table 1:** MSE Scale.

Indicators of Mental Health	Sub-category or components of mental health indicator	Normal	Impaired	Method
**Appearance**	Normal, anxious, depressive, elated and irritated	Normal appearance	Anxious, depressive, elated and irritated	Facial expression and conversation
**Attitude**	Cooperative, friendly, attentive, interested, frank and defensive	Cooperative, friendly, attentive, interested, frank	Defensive state	Respondent’s reaction during interview
**Behavior**	Mannerism, gesture, stereotype, flexibility, hyperactivity, hypoactivity, rigidity, and agility	Gesture, flexibility, agility	Mannerism, stereotype, hyperactivity, hypoactivity and rigidity	Conversation and observation
**Mood**	Euthymic, depressive, dysphoric, irritable, elevated, and euphoric	Euthymic	Depressive, dysphoric, irritable, elevated, and euphoric	Judged by facial expression, posture, and movement
**Sleep**	Normal, insomnia and hypersomnia	Normal	Insomnia and hypersomnia	By asking question
**Clarity of Speech**	Clear, slurred, lisping, relevant and incoherent	Clear, relevant	Slurred, lisping, incoherent	Conversation
**Attention**	Sufficient, deficient, easily distractible, short span of attention, poor or adequate concentration, and preoccupation	Sufficient, preoccupation	Deficient, easily distractible, short span of attention, poor or inadequate concentration	Observation of activities throughout the survey
**Memory**	Remote, recent, recent past, and immediate memory	Those who memorized	Those who were unable to memorize	By asking some questions related to her daily activities, location of everyday items, notable events and repeating some questions.
**Insight**	Intellectual, true insight, and Impaired insight	Intellectual, true insight	Impaired insight	By asking questions to participant about her thinking.
**Judgment**	Critical, automatic, and impaired judgment	Critical, automatic judgment	impaired judgment	Participant’s thinking

### 2.6 Data collection

The interviews were conducted in person, and a clinical examination (MSE scale) was substituted with a pilot-tested questionnaire. The questionnaire and checklist were created based on selected variables related to the MSE scale, and we scored impaired intellectual status to normal mental status for every indicator of intellectual fitness within the MSE (**Table 1**). Moreover, we collected health and well-being information as independent variables.

### 2.7 Statistical analysis

We applied generalized linear models (GLM) to conduct unadjusted and adjusted analyses using robust standard errors to account for correlated data. When examining mental health status, we considered variables such as impaired and normal. In our study, we thoroughly examined various confounding factors that could potentially influence the effects being analyzed. We applied the log link function g(u)=ln(u) within generalized linear models (GLMs) to model the relationship between the expected value of the outcome (u) and a linear combination of predictors. The GLM with log link provided a robust framework for estimating PR (41).

Independent variables were selected based on existing theories or evidence suggesting their potential relationship with the dependent variable. These factors included the respondent’s age, education level, spouse’s education, household income, health status, experiences of domestic violence, family disharmony, social security, and support. These factors were presumed to impact both the dependent variable and the independent variables of interest in our research. We included all covariates that showed a significant association (p<0.2) with the dependent variable in the unadjusted multivariate analyses.

A comprehensive literature review was conducted to identify variables that may act as potential confounders impacting the dependent variables of interest (42). Adjusting for confounders is essential for a robust and reliable analysis. Some variables like physical health involve self-reported, which can significantly impact outcomes, potentially distorting the true relationships between key variables. We applied the Generalized Linear Model (GLM) method to assess each independent variable in relation to the dependent variables, systematically identifying potential confounders for distinct outcomes. By incorporating statistical controls, our analysis achieved greater precision and more accurately represented the genuine associations present in the data. For example, physical health may independently influence psychological outcomes, while family disharmony can shape results through various relational and social factors. Addressing these confounders clarifies their role, ensuring that the findings accurately isolate the effects of the primary variables.

### 2.8 Ethical consideration

In contexts like Bangladesh, cultural sensitivities around mental health topics are key to demonstrating ethical transparency where stigma around mental health is prevalent. The investigator approached conversations with empathy, using culturally appropriate language to reduce discomfort and avoid reinforcing stigma. Providing reassurance about confidentiality and the non-judgmental nature of the study is crucial in building trust. Additionally, being aware of and respecting cultural norms, the investigator ensured that participants felt safe and respected throughout the interview process.

Prior to every interview, written consent was obtained from all respondents, who willingly agreed to participate. Each participant received comprehensive information regarding the study’s objectives, significance, and purpose, ensuring transparency and informed engagement throughout the research process. In addition, ethical approval was taken dated 12 August 2015 (NIPSOM/IRB/2015) from the Institutional Review Board of the National Institute of Preventive and Social Medicine (NIPSOM). The mental state was evaluated by administering a pilot-tested questionnaire during the examination of mental health.

## 3 Results

### 3.1 Socio-demographic and social influencing factors among women in rural Bangladesh

Among the 401 women participating in the survey, the average age was 29 years (SD=7.25) and 58% were below 29. Forty-two percent of the participants had completed primary education, and the majority (94%) were homemakers. A sizable portion of spouses (42%) reported being illiterate. A considerable proportion of respondents (37%) were professional employees or day laborers. Additionally, 27% were traders, 25% were farmers, and 12% were service holders. A considerable proportion of families (59%) reported a monthly income of less than USD 125. Among the participants, 39% indicated poor physical fitness. and 73% reported experiencing family disharmony and domestic violence. The percentage of individuals experiencing a loss of social safety was 21%. Sixty-nine percent of individuals found who were facing a reduction in social assistance (**Table 2**).

**Table 2:** Socio-demographic and social influencing factors among women in rural Bangladesh (N=401).

Indicators	Frequency (n)	Percentage (%)
**Age of respondents** <29 years > 29 years	234 167	58 42
**Respondents Education** No education Up to Primary Secondary and above	102 170 129	25 42 32
**Respondents Occupation** Housewife Service/business Skilled worker Labor	375 11 9 6	94 3 2 1
**Education of Spouse** No education Up to Primary Secondary and above	167 132 102	42 33 25
**Occupation of Spouse** Farmer/cultivator Traders/business Skilled worker/Day labor Service holder	100 108 146 46	25 27 37 12
**Household income (**USD) < $125 $ 125-250 >$ 250	238 123 40	59 31 10
**Ownership of living house** Self-owned Rent/government land	392 9	98 2
**Health and wellbeing** Good Poor	245 156	61 39
**Home violence** Very often Rarely	291 110	73 27
**Family disharmony** Present Absent	291 110	73 27
**Social security** Yes No	315 86	79 21
**Social support** Yes No	124 277	31 69

The overall prevalence of mental health issues was 26%. Specifically, 32% of the participants had problems related to appearance, 45.1% had attitude issues, and 43.9% found behavioral problems. Nearly forty percent of the participants disclosed mood disorders, while 23.4% reported sleep disorders, respectively. Around ten percent exhibited speech defects, 23.4% observed attention issues, and 23.2% had memory problems. Poor insight was noted in 10.7% of the participants, while 8.5% had judgment issues (**Supplementary Table S1**).

Based on the association with independent variables to certain domains of MSE scale, we found women of both age groups had a similar prevalence of impaired behavior, insight, and judgment. Impairments in mood and memory were more prevalent among the older age group compared to younger counterparts, with rates of 43% and 30%, respectively. Women with no education exhibited 52% of impaired behavior, while those with primary education had a slightly lower at 48%compared to those with secondary education and above. However, 46% mood impairment was observed in the group belonging to primary education, and 29% impaired memory was found in secondary education and above group. Among housewives, 24% exhibited impaired memory compared to others. In contrast, 56% of skilled workers found impaired behavior. Women employed as laborers exhibited notable impairments, with 83% experienced mood disturbances and 17% showed impairments in both insight and judgment. Fifty-two percent of women whose spouses had no education demonstrated impaired behavior, On the other hand, among 46% of respondents whose spouses had attained secondary education or higher, exhibited impaired mood, 27% showed memory impairment, 11% had impaired insight, and 10% demonstrated impaired judgment. Women from low households income group (<125 USD) found 48% of behavioral problems. Conversely, individuals from middle and relatively higher-income group reported 44% and 45% of impaired mood. However, 29% of the middle-income group experienced memory impairment, and 13% showed impaired insight, compared to relatively higher-income group (**Supplementary Table S2**).

Regarding social influencing factors, 48% of women in good health were found to exhibit impaired behavior, compared to those in poor health. Women with poor health conditions exhibited higher impairments across several domains, including mood 49%, memory 28%, insight 15%, and judgment 9% (**Supplementary Table S2**).

Forty-eight percent of women who experienced frequent home violence reported impaired behavior. Interestingly, even women who rarely experienced home violence showed similar notable impairments in multiple domains including mood 44%, memory 25%, insight 11%, and judgment 9%. A similar pattern of impairments was observed among women experiencing family disharmony (**Supplementary Table S2**).

While examining social security, both those with and without social security exhibited comparable rates of behavioral impairment, 44% and 43% respectively. Among women without social security, impairments were noted in 44% of mood, 17% of insight, and judgment. In contrast, women with social security exhibited a 24% rate of memory impairment. In terms of social support, both groups with and without support demonstrated similar rates of behavioral and mood impairments. However, 15% of women in the support group showed impaired insight and 13% in judgment, while 25% of women without support observed memory impairment (**Supplementary Table S2**).

### 3.2 Factors associated with impaired mental health among women in rural Bangladesh

#### 3.2.1 Unadjusted analysis

We found that women’s age, education, occupation, spouse education, and household income were associated with mental health outcomes in the unadjusted analysis. The prevalence of behavioral problems was lower in respondents with secondary education or higher compared to those with no education (PR = 0.62, 95% CI: 0.46, 0.85). Additionally, respondents’ impaired behavior was less likely to be associated with higher education of their spouse (PR = 0.63, 95% CI: 0.46, 0.86) (**Table 3**).

**Table 3:** Factors associated with impaired mental health among women in rural Bangladesh.

Characteristics	N	Behavior			Mood			Memory			Insight			Judgment		
		n (%)	Unadjusted^a^ PR, 95% CI	Adjusted^b^ PR, 95% CI	n(%)	Unadjusted^a^ PR, 95% CI	Adjusted^b^ PR, 95% CI	n (%)	Unadjusted^a^ PR, 95% CI	Adjusted^b^ PR, 95% CI	n (%)	Unadjusted^a^ PR, 95% CI	Adjusted^b^ PR, 95% CI	n (%)	Unadjusted^a^ PR, 95% CI	Adjusted^b^ PR, 95% CI
Socioeconomics and demographics																
**Age of respondents<29 years> 29 years**	234 167	103 (44) 73 (43)	Ref 0.99 (0.79, 1.24)	Ref 1.09 (0.88, 1.35)	88(38) 72 (43)	Ref 1.15 (0.90, 1.46)	Ref 1.07 (0.84, 1.37)	43 (18) 50 (30)	Ref 1.63 (1.14, 2.33)	Ref 1.52 (1.06, 2.18)	25 (11) 18 (11)	Ref 1.01(0.57, 1.79)	Ref 0.96(0.54, 1.72)	21 (9) 13 (8)	Ref 0.87 (0.45,1.68)	–
**Respondents Education** No education Up to primary Secondary and above	102 170 129	53 (52) 81 (48) 42 (33)	Ref 0.92 (0.72,1.17) 0.62 (0.46, 0.85)	Ref 0.95 (0.74, 1.22) 0.73 (0.52, 1.00)	29 (28) 79 (46) 52 (40)	Ref 1.63 (1.15,2.31) 1.42 (0.98, 2.06)	Ref 1.56 (1.09,2.23) 1.28 (0.84, 1.94)	20 (20) 36 (21) 37 (29)	Ref 1.08 (0.67,1.76) 1.40 (0.87,1.76)	Ref 1.08 (0.67,1.76) 1.40 (0.87,2.27)	13 (13) 18 (11) 12 (9)	Ref 0.83(0.43,1.62) 0.73(0.35,1.53)	Ref 0.83(0.43,1.62) 0.77(0.37,1.61)	11(11) 12 (7) 11 (9)	Ref 0.65(0.29,1.43) 0.79(0.36,1.75)	Ref 0.66 (0.31,1.41) 0.87 (0.41, 1.94)
**Respondents Occupation** Housewife Service/business Skilled worker Labor	375 11 9 6	167 (45) 3 (27) 5 (56) 4 (17)	Ref 0.62 (0.23,1.62) 1.25 (0.69,2.26) 0.37 (0.06,2.25)	–	146 (39) 5 (45) 4 (44) 5 (83)	Ref 1.17 (0.60, 2.26) 1.14 (0.54, 2.39) 2.14 (1.46, 3.13)	–	90 (24) 0 (0) 2 (22) 1 (17)	Ref -^*^ 0.93 (0.27, 3.18) 0.69 (0.12, 4.19)	–	41 (11) 1 (9) 0 (0) 1 (17)	Ref 0.83(0.13, 5.51) -^*^ 1.52(0.25, 9.34)	–	31 (8) 1 (9) 1 (11) 1 (17)	Ref 1.09 (0.16, 7.34) 1.34 (0.21, 8.79) 2.02 (0.33, 12.45)	–
**Education of Spouse** No education Up to Primary Secondary and above	167 132 102	86 (52) 57 (43) 33 (32)	Ref 0.84 (0.66,1.07) 0.63 (0.46, 0.86)	Ref 0.88 (0.69,1.13) 0.73 (0.52, 1.03)	59 (35) 54 (41) 47 (46)	Ref 1.16 (0.87,1.55) 1.30 (0.98,1.75)	Ref 1.12 (0.82, 1.51) 1.19 (0.83, 1.69)	44 (26) 21 (16) 28 (27)	Ref 0.60 (0.38,0.96) 1.04 (0.69,1.56)	Ref 0.53 (0.33,0.85) 0.81 (0.51,1.27)	17 (10) 15 (11) 11 (11)	Ref 1.12(0.58,2.15) 1.06(0.52,2.17)	Ref 1.12(0.58,2.15) 1.05(0.52, 2.16)	14 (8) 10 (9) 10(10)	Ref 0.90 (0.41, 1.97) 1.17 (0.54, 2.53)	–
**Household income(USD)** 125 125-250 >250	238 123 40	115 (48) 52 (42) 9 (23)	Ref 0.87 (0.68, 1.12) 0.47 (0.26, 0.84)	Ref 0.93 (0.73, 1.19) 0.48 (0.27, 0.86)	88 (37) 54 (44) 18 (45)	Ref 1.19 (0.92, 1.54) 1.22 (0.83, 1.78)	Ref -^*^ 0.84 (0.63, 1.13)	48 (20) 36 (29) 9 (23)	Ref 1.45 (0.99, 2.11) 1.12 (0.59, 2.09)	Ref 1.36 (0.93, 1.97) 1.05 (0.57, 1.96)	23 (10) 17 (13) 3 (7)	Ref 1.43(0.79, 2.57) 0.77(0.44, 2.46)	Ref 1.32(0.74, 2.38) 0.77(0.24, 2.41)	23 (10) 9 (7) 2 (5)	Ref 0.78 (0.36, 1.59) 0.52 (0.13, 2.11)	Ref 0.74 (0.36, 1.52) 0.58 (0.14, 2.35)
Health and well-being on Mental Health																
**Physical condition** Good Poor	245 156	118 (48) 58 (37)	Ref 0.77 (0.61, 0.98)	Ref 0.77 (0.61, 0.98)	84 (34) 76 (49)	Ref 1.42 (1.12, 1.80)	Ref 1.42 (1.13, 1.79)	49 (20) 44 (28)	Ref 1.41 (0.99, 2.01)	Ref 1.32 (0.92, 1.89)	20 (8) 23 (15)	Ref 1.95(1.03, 3.68)	Ref 1.73(0.96, 3.11)	20 (8) 14 (9)	Ref 1.11 (0.54, 2.27)	Ref 0.94 (0.49, 1.80)
**Home violence** Almost never Very often	110 291	42 (38) 134 (46)	Ref 1.21 (0.92, 1.58)	Ref 1.23 (0.95, 1.61)	48 (44) 112 (38)	Ref 0.88 (0.68, 1.14)	Ref 0.72 (0.53, 0.98)	28 (25) 65 (22)	Ref 0.88 (0.59, 1.29)	Ref 0.93 (0.63, 1.38)	12 (11) 31 (11)	Ref 0.97(0.48, 1.97)	Ref 0.85(0.45, 1.62)	10 (9) 24 (8)	Ref 0.89 (0.42, 1.95)	Ref 0.75 (0.37, 1.54)
**Family disharmony** Absent Present	110 291	45 (41) 131(45)	Ref 1.10 (0.85, 1.42)	Ref 1.11 (0.86, 1.44)	56 (51) 104 (36)	Ref 0.70 (0.55, 0.89)	Ref 0.70(0.56, 0.89)	19 (17) 74 (25)	Ref 1.47 (0.94, 2.32)	Ref 1.40 (0.88, 2.21)	16 (15) 27 (9)	Ref 0.60(0.31, 1.16)	Ref 0.75(0.36, 1.57)	12 (11) 22 (8)	Ref 0.67 (0.32, 1.40)	Ref 1.25 (0.59, 2.68)
**Social security** Yes No	315 86	139 (44) 37 (43)	Ref 0.97 (0.74, 1.28)	Ref .96 (0.62, 1.49)	122 (39) 38 (44)	Ref 1.14 (0.87, 1.50)	Ref 0.74 (0.52, 1.05)	75 (24) 18 (21)	Ref 0.88 (0.58, 1.39)	Ref 1.04 (0.62, 1.75)	28 (9) 15 (17)	Ref 2.17(1.10, 4.27)	Ref 1.51(0.71, 3.22)	19 (6) 15 (17)	Ref 3.29 (1.59, 6.79)	–
**Social support** Yes No	124 277	123 (44) 53 (43)	Ref 0.96 (0.76, 1.23)	Ref 0.90 (0.71, 1.15)	110 (40) 50 (40)	Ref 1.02 (0.78, 1.32)	Ref 0.72(0.53, 1.97)	70 (25) 23 (19)	Ref 0.73 (0.48, 1.12)	Ref 0.85 (0.52, 1.38)	24 (9) 19 (15)	Ref 1.90(1.00, 3.63)	Ref 1.61(0.81, 3.18)	18 (7) 16 (13)	Ref 2.13 (1.05, 4.34)	–

PR: CI: Confidence interval.

a We determined the PR by using a generalized linear model (GLM).

b Adjusted for respondent’s age, occupation, occupation of spouse, ownership of household, physical condition, home violence, family disharmony, social security and social support.*In adjusted analysis, we found some cell values less than 5. So, we have blank cells for adjusted result.

Furthermore, we observed that a higher household income exhibited a comparatively weaker association with impaired behavior (PR = 0.47 [0.26, 0.84]). Impaired mood was notably more prevalent among individuals with primary education (PR = 1.63 [1.15, 2.31]), as well as those employed in labor (PR = 2.14 [1.46, 3.13]). Respondents aged over 29 years were 1.63 times more likely to experience impaired memory (1.14, 2.33). Moreover, impaired memory showed a less pronounced association with respondents whose spouse had primary education (PR = 0.60 [0.38, 0.96]), as well as with occupation of spouse (PR = 0.57 [0.34, 0.97]) (**Table 3**).

The mental health outcomes of respondents were notably associated with their physical condition, family dynamics, social security, and social support. Those with poor physical health were less likely to exhibit impaired behavior (PR = 0.77 [0.61, 0.98]), yet more prone to experiencing impaired mood (PR =1.42 [1.12, 1.80]) and insight 1.95 [1.03, 3.68]. Interestingly, family disharmony exhibited a lower likelihood of association with mood (PR = 0.70 [0.55, 0.89]). However, impaired insight and judgment were significantly more associated with social security compared to mood (PR = 2.17 [1.10, 4.27] and (PR = 3.29 [1.59, 6.79]), respectively. Moreover, social support displayed a higher association with impaired insight (PR =1.90 [1.00, 3.63]) and judgment (PR = 2.13 [1.05, 4.34]) (**Table 3**).

#### 3.2.2 Adjusted analysis

After controlling for potential confounders in multivariable analysis, we found that household income was significantly associated with behavioral problems, which aligns with the findings from the unadjusted analysis. The presence of impaired mood was almost twice as likely among respondents with primary education (PR= 1.56; 95% CI 1.09, 2.23), and this association remained significant even after adjusting for physical condition and family disharmony. Memory impairment was significantly associated with the age group >29 (PR= 1.52; 95% CI 1.06, 2.18), while the association with spouse’s primary education was less significant (PR= 0.53; 95% CI 0.33, 0.85) (**Table 3**). Respondents physical health, and family disharmony were significantly associated with mood disorder (PR= 1.42; 95% CI 1.13, 1.79), (PR= 0.70; 95% CI 0.56, 0.89) (**Table 3**).

## 4 Discussion

This study explores the socio-demographic determinants of mental health disorders among rural women in the Mymensingh district of Bangladesh, identifying significant associations with factors such as age, education, household income, spousal education, and exposure to domestic violence. These findings are consistent with prior research in LMICs, underscoring the influence of structural inequalities on mental health outcomes and highlighting the urgent need for targeted, context-specific interventions.

### 4.1 Age as a vulnerability factor

Younger women exhibited greater mental health impairments, highlighting their vulnerability to socio-economic stressors such as unemployment, marital conflicts, and limited coping skills. These results parallel studies in metanalysis suggest, that younger populations are at increased risk of anxiety and depression due to unstable social networks and inadequate life experiences (43)

### 4.2 Education and socioeconomic status as critical determinants

The demographic and socioeconomic context of our participants in rural Bangladesh offers insight into the intricate dynamics shaping women’s mental health. Education and household income were found to be pivotal in shaping mental health outcomes. Women with higher educational attainment demonstrated better mental health, likely due to increased mental health literacy, improved coping mechanisms, and greater decision-making autonomy (12). Similarly, higher household income alleviates financial stressors, reduces food insecurity, and facilitates access to healthcare, as corroborated by studies in Kenya, India, and Nigeria (16, 44, 45). Research in Nepal and Uganda further supports the role of economic empowerment in reducing mental health symptoms, particularly among rural women (46, 47). However, higher education does not entirely eliminate mental health challenges, as stressors like economic instability and family pressures can still contribute to impairments (48). The relationship between income and mental health is complex reflecting the stress of financial hardship and also suggesting vulnerabilities persist despite some financial stability (49). Studies in Zimbabwe and Ethiopia have found that socio-demographic factors like food insecurity, intimate partner violence, and lack of social support significantly impact rural women’s mental health (50, 51).

### 4.3 Impact of domestic violence

Domestic violence emerged as a significant factor contributing to mental health impairments, with affected women displaying higher rates of mood, memory, and judgment issues. These findings align with study, which report strong correlations between intimate partner violence and increased rates of depression and anxiety (52). Women experiencing frequent home violence (53) and who rarely experienced home violence exhibited significant comparable impairments reinforcing the connection between family conflict and mental health deterioration. Similar patterns are observed in Sub-Saharan Africa, where exposure to violence intensifies stress responses, fostering a cyclical relationship between vulnerability and mental health impairment (54, 55). Fear and stigma often deter women from seeking help, compounding the problem (56).

### 4.4 Role of spousal education, and family dynamics

Spousal education positively influenced mental health outcomes, a trend consistently with findings from Mexico and Pakistan (57, 58). Educated spouses likely provide emotional and financial support, buffering the adverse effects of socio-economic stressors. Additionally, family disharmony, often tied to domestic violence, was linked to impaired mood and judgment, similar to findings in Turkey where family support is critical to mitigating mental health challenges (59). A participatory mental health initiative in Nepal demonstrated that traditional gender roles and local power structures significantly impact mental health outcomes and access to services beyond socioeconomic status (60). Gender expectations and masculinities may also play an important role in gender disparities in mental health such as domestic violence, sexual abuse, unpaid caring work, higher hours of work, low social status, and lack of access to reproductive rights and education that move beyond standard socio-demographics (61). Notably, family disharmony and domestic violence are linked to mental health outcomes, a concern given Bangladesh’s high prevalence of intimate partner violence. Cultural acceptance of violence, particularly in rural areas, underscores the need for tailored community interventions to address mental health challenges effectively (62). The study findings highlight the significant impact of family disharmony on the behavior and mood of respondents, consistent with previous research (52, 63). Women in Bangladesh frequently shoulder the responsibility of ensuring their family’s welfare amidst poverty, facing additional pressures in securing basic necessities (64).

The relationship between physical health and mental health outcomes underscores their interdependence. Women in poor health demonstrated compounding mental health impairments. Conversely, women in good health exhibited notable behavioral impairments possibly due to unaddressed psychological stressors unrelated to physical health (65) highlighting the bidirectional nature of the physical and mental health relationship.

The findings on social security and support highlight the complex relationship between social resources and mental health. Behavioral impairments were similar among women with and without social security suggesting that factors like healthcare quality may play a more significant role than financial security alone (12). Social security systems in rural areas are often underdeveloped, creating significant barriers to accessing necessary benefits. This lack of support profoundly impacts the mental health of rural women, leading to financial stress, limited healthcare access, and increased social isolation. Gender discrimination and limited opportunities further compromise their well-being (66). Cultural barriers, such as the perception of mental illness as fate in rural areas, hinder acknowledgment and awareness of mental health issues as fundamental human rights. The stigma surrounding mental health further discourages seeking help or discussing struggles openly (52, 63).

### 4.5 Causality and bidirectional relationships

Although the cross-sectional design limits the ability to establish definitive causation, the strong alignment of these findings with prior global studies provides compelling evidence for causal links between socio-demographic factors and mental health outcomes. For example, education has been shown to enhance resilience and improve problem-solving skills, equipping individuals with better-coping mechanisms for mental health challenges (67), Socioeconomic resources can improve mental health outcomes by enhancing access to healthcare and reducing life stressors. Individuals with more financial resources are often better able to afford mental health services, medications, and other forms of treatment, increasing their likelihood of receiving timely and effective care (68). Additionally, socioeconomic stability can reduce stress related to basic needs like housing, food, and financial security, which are major contributors to mental distress. Lower stress levels and stable resources can foster resilience and provide a buffer against mental health challenges, creating a healthier environment that supports physical and psychological well-being (69). Nevertheless, the potential for reverse causality must be acknowledged: individuals with impaired mental health may face greater difficulty in pursuing education or securing stable income due to cognitive or emotional impairments (46). These bidirectional relationships highlight the complexity of socio-demographic determinants and underscore the need for comprehensive, multi-pronged interventions. Addressing both the root causes and the consequences of poor mental health will yield more sustainable outcomes (49). This emphasizes the importance of integrating mental health care into broader socio-economic development initiatives.

The prevalence of mental health issues in Bangladesh, coupled with cultural stigma and limited healthcare resources, presents a significant challenge for mental health services. Rehabilitation centers are scarce, with notable ones like the National Mental Health Institute in Dhaka and a 500-bed mental hospital in Hemayetpur, Pabna district. While there are 50 outpatient mental health facilities, only a small fraction caters to children and adolescents. Additionally, there are 31 community-based psychiatric inpatient units. However, primary healthcare providers’ exposure to mental health remains limited, with only 21-50% having yearly interactions. Addressing this gap requires expanding mental health services and enhancing integration into primary care (62).

Our research underscores the need for comprehensive interventions to enhance women’s mental health in rural Bangladesh. These interventions should account for educational, socioeconomic, and cultural influences on mental well-being. Culturally sensitive, community-based mental health programs addressing women’s specific challenges are essential. The inclusion of mobile applications in psychological crisis intervention represents a promising approach to addressing mental health issues, particularly in settings where access to traditional face-to-face therapy may be limited or users who feel uncomfortable with in-person counseling.

In low- and middle-income countries (LMICs), community-based mental health interventions have proven effective by adopting task-shifting strategies, where non-specialist health workers or community members deliver mental health services. The Friendship Bench in Zimbabwe trains lay health workers to provide talk therapy for depression and anxiety, enhancing accessibility and acceptance within local communities (70). Similarly, Pakistan’s Thinking Healthy Program integrates cognitive behavioral therapy into maternal health care, which benefits for maternal mental health outcomes (71). In India, the Atmiyata project empowers rural volunteers to provide mental health support and reduce stigma, fostering community-based mental health resilience (72). Strengthening the capabilities of primary healthcare providers to recognize and treat mental health issues could be a significant step towards improving the mental health landscape in Bangladesh. Moreover, addressing demand-side barriers is essential, including patient education, community empowerment, and advocacy, which are vital tools for overcoming these challenges.

### 4.6 Internal and external validity

This study benefits from a strong design framework, including multistage sampling, the use of a standardized Mental State Examination (MSE) scale, and well-defined data collection protocols. These elements enhance the internal validity by minimizing selection bias and ensuring the reliability of mental health assessments. Furthermore, the rigorous training of investigator and consistent monitoring during data collection reduced potential inconsistencies (73). However, self-reported data introduces the risk of social desirability bias, especially in a cultural context where mental health stigma remains pervasive (74). While confidentiality measures likely reduced this bias, the cultural context may still have influenced participants’ willingness to report symptoms accurately. Addressing this limitation in future research through triangulated methods, such as clinical interviews or independent observations, could further strengthen validity.

The findings of this study are broadly applicable to rural populations in LMICs, where similar socio-economic, educational, and healthcare disparities exist. Previous studies from regions such as South Africa and Southeast Asia have similarly identified socio-demographic factors as key determinants of mental health outcomes, lending support to the generalizability of these results (75, 76). However, variations in cultural norms, the degree of mental health stigma, and the availability of healthcare services may limit direct applicability to non-Bangladesh contexts. For example, differing levels of gender-based inequality or access to community support structures may influence how mental health outcomes manifest (77). Therefore, while the findings provide valuable insights for LMIC contexts, localized adaptations will be necessary to develop contextually appropriate interventions.

### 4.7 Strengths and limitations

The study explores the strength and importance of investigating the range of factors that influence the mental well-being of women residing in rural areas of Bangladesh. This cross-sectional study aims to shed light on the unique challenges faced by women in these communities and the potential implications for their mental health. By examining the social, cultural, and economic factors that contribute to mental health outcomes, this research seeks to provide valuable insights into the specific needs and interventions required to support the mental well-being of women in rural Bangladesh. Through a comprehensive analysis of the data collected, this study aims to contribute to the existing body of knowledge on mental health in rural communities and inform future policies and programs aimed at improving the mental health outcomes of women in Bangladesh.

The study has several limitations: First, the cross-sectional design prevents the establishment of temporal or causal relationships between socio-demographic variables and mental health outcomes. Future studies employing longitudinal designs could better clarify the direction of these relationships. Second, the MSE scale, while robust, excluded cognitive and perceptual domains that may have provided a more comprehensive assessment of mental health functioning. Incorporating these domains in future studies would allow for a more holistic understanding of mental health impairments. Third, stigma surrounding mental illness likely led to underreporting of symptoms, particularly for mood and behavioral disorders, potentially underestimating the true prevalence of mental health issues. Lastly, the exclusion of remote and hard-to-reach areas due to logistical constraints may have resulted in the underrepresentation of the most vulnerable populations, thereby limiting the comprehensiveness of the findings. Future research should aim to address these gaps through broader sampling, longitudinal approaches, and the inclusion of underrepresented populations to improve the robustness and generalizability of the conclusions.

## 5 Conclusions

This study highlights the significant influence of socio-demographic factors, including education, income, spousal support, and experiences of domestic violence, on mental health functioning among rural women in Bangladesh. The findings underscore the urgent need for culturally tailored interventions to address mental health disparities in these communities. By integrating mental health services with broader socio-economic initiatives, improving resource allocation, and fostering intersectoral collaboration, policymakers can effectively reduce mental health burdens and promote holistic well-being in rural Bangladesh. Future studies should prioritize longitudinal studies that track mental health trajectories over time and include more diverse demographic groups to enrich our understanding. Emphasizing the importance of broader sampling and a deeper examination of socio-demographic factors would underscore the study’s value and provide direction for future research. It is crucial for government to prioritize counseling and awareness-building programs to tackle this problem effectively.

Culturally tailored community-based mental health programs are crucial to reducing stigma and improving mental health literacy in rural Bangladesh. Stigma remains a significant barrier to accessing mental health care, as reported in studies on Bangladeshi rural populations (6). Programs should focus on educating communities about the signs and symptoms of mental health issues while challenging misconceptions surrounding mental illness. For example, interventions like the Shasthya Shebika program, which leverages local health volunteers to promote maternal and child health, could be adapted to include mental health education and counseling (78). Addressing domestic violence as a critical factor in mental health impairments is also vital. Policies should include legal frameworks and community-based initiatives to protect women and provide support services, as highlighted by recent gender-based violence interventions in rural districts (79).

## Data Availability

The original contributions presented in the study are included in the article/**Supplementary Material**. Further inquiries can be directed to the corresponding author.
