# Supplementary material for: Socio-demographic factors associated with mental health disorders among rural women in Mymensingh, Bangladesh
Source: Front Psychiatry. 2025 Feb 18;16:1446473. doi: 10.3389/fpsyt.2025.1446473 (PMC11876128; doi:10.3389/fpsyt.2025.1446473)
Supplement: Supplementary file 1 [file Table1.docx]

**Supplementary tables**

**Supplementary Table 1: Assessment of Mental Health by using MSE Scale (n=401)**

| **Variables** | **Mental Health Status** | | | |
| --- | --- | --- | --- | --- |
|  | **Normal** | | **Impaired** | |
|  | **Frequency** | **Percentage** | **Frequency** | **Percentage** |
| Appearance | 272 | 67.8 | 129 | 32.2 |
| Attitude | 220 | 54.9 | 181 | 45.1 |
| Behavior | 225 | 56.1 | 176 | 43.9 |
| Mood | 241 | 60.1 | 160 | 39.9 |
| Sleep | 307 | 76.6 | 94 | 23.4 |
| Speech | 362 | 90.3 | 39 | 9.7 |
| Attention | 307 | 76.6 | 94 | 23.4 |
| Memory | 308 | 76.8 | 93 | 23.2 |
| Insight | 358 | 89.3 | 43 | 10.7 |
| Judgment | 367 | 91.5 | 34 | 8.5 |
| **Average** | **296.7** | **74.0** | **104.3** | **26.0** |

**Supplementary Table 2: Socioeconomic Status and Mental health among rural women**

| **Characteristics** | **N** | **Behavior*** | **Mood*** | **Memory*** | **Insight*** | **Judgment*** |
| --- | --- | --- | --- | --- | --- | --- |
|  |  | **n (%)** | **n (%)** | **n (%)** | **n (%)** | **n (%)** |
| **Age of respondents**  **<29 years**  **> 29 years** | 234  167 | 103 (44)  73(43) | 88 (38)  72 (43) | 43 (18)  50 (30) | 25 (11)  18 (11) | 21 (9)  13 (8) |
| **Respondents Education**  No education  Up to Primary  Secondary and above | 102  170  129 | 53 (52)  81 (48)  42 (33) | 29 (28)  79 (46)  52 (40) | 20 (20)  36 (21)  37 (29) | 13 (13)  18 (11)  12 (9) | 11 (11)  12 (7)  11 (9) |
| **Respondents Occupation**  Housewife  Service/business  Skilled worker  Labor | 375  11  9  6 | 167 (45)  3 (27)  5 (56)  4 (17) | 146 (39)  5 (45)  4 (44)  5 (83) | 90 (24)  0 (0)  2 (22)  1 (17) | 41 (11)  1 (9)  0 (0)  1 (17) | 31 (8)  1 (9)  1 (11)  1 (17) |
| **Spouse education**  No education  Up to primary  Secondary and above | 167  132  102 | 86 (52)  57 (43)  33 (32) | 59 (35)  54 (41)  47 (46) | 44 (26)  21 (16)  28 (27) | 17 (10)  15 (11)  11 (11) | 14 (8)  10 (9)  10 (10) |
| **Household income**  <125  125-250  >250 | 238  123  40 | 115 (48)  52 (42)  9 (23) | 88 (37)  54 (44)  18 (45) | 48 (20)  36 (29)  9 (23) | 23 (10)  17 (13)  3 (7) | 23 (10)  9 (7)  2 (5) |
| **Social influencing factors of Mental Health** | | | |  | | |
| **Physical condition**  Good  Poor | 245  156 | 118 (48)  58 (37) | 84 (34)  76 (49) | 49 (20)  44 (28) | 20 (8)  23 (15) | 20 (8)  14 (9) |
| **Home violence**  Almost never  Very often | 110  291 | 134 (46)  42 (38) | 112 (38)  48 (44) | 65 (22)  28 (25) | 31 (11)  12 (11) | 24 (8)  10 (9) |
| **Family disharmony**  Absent  Present | 110  291 | 45 (41)  131(45) | 56 (51)  104 (36) | 19 (17)  74 (25) | 16 (15)  27 (9) | 12 (11)  22 (8) |
| **Social security**  Yes  No | 315  86 | 139 (44)  37 (43) | 122 (39)  38 (44) | 75 (24)  18 (21) | 28 (9)  15 (17) | 19 (6)  15 (17) |
| **Social support**  Yes  No | 124  277 | 123 (44)  53 (43) | 110 (40)  50 (40) | 70 (25)  23 (19) | 24 (9)  19 (15) | 18 (7)  16 (13) |

*Results shows impaired mental health in dependent variables
